# Supplementary material for: Dialysate and plasma meropenem concentrations in continuous intraperitoneal regimen during peritoneal-dialysis-related peritonitis
Source: PLoS One. 2025 Jan 6;20(1):e0312160. doi: 10.1371/journal.pone.0312160 (PMC11703071; doi:10.1371/journal.pone.0312160)
Supplement: S1 File — (DOCX) [file pone.0312160.s001.docx]

**S1: Sample size calculation**

The primary objective of this study was to estimate plasma and dialysate meropenem level after continuous regimen of intraperitoneal meropenem administration in patient with PD related peritonitis.

Regard to the formula for estimating an infinite population mean, the minimum sample size would be calculated using the following equation and parameters. According to the literature review, the mean areas under the curve from 0 to 24 hours (AUC_0–24_) of meropenem in serum which will be provided adequate plasma meropenem were 173.5 ± 29.7 mg· h/liter [10].


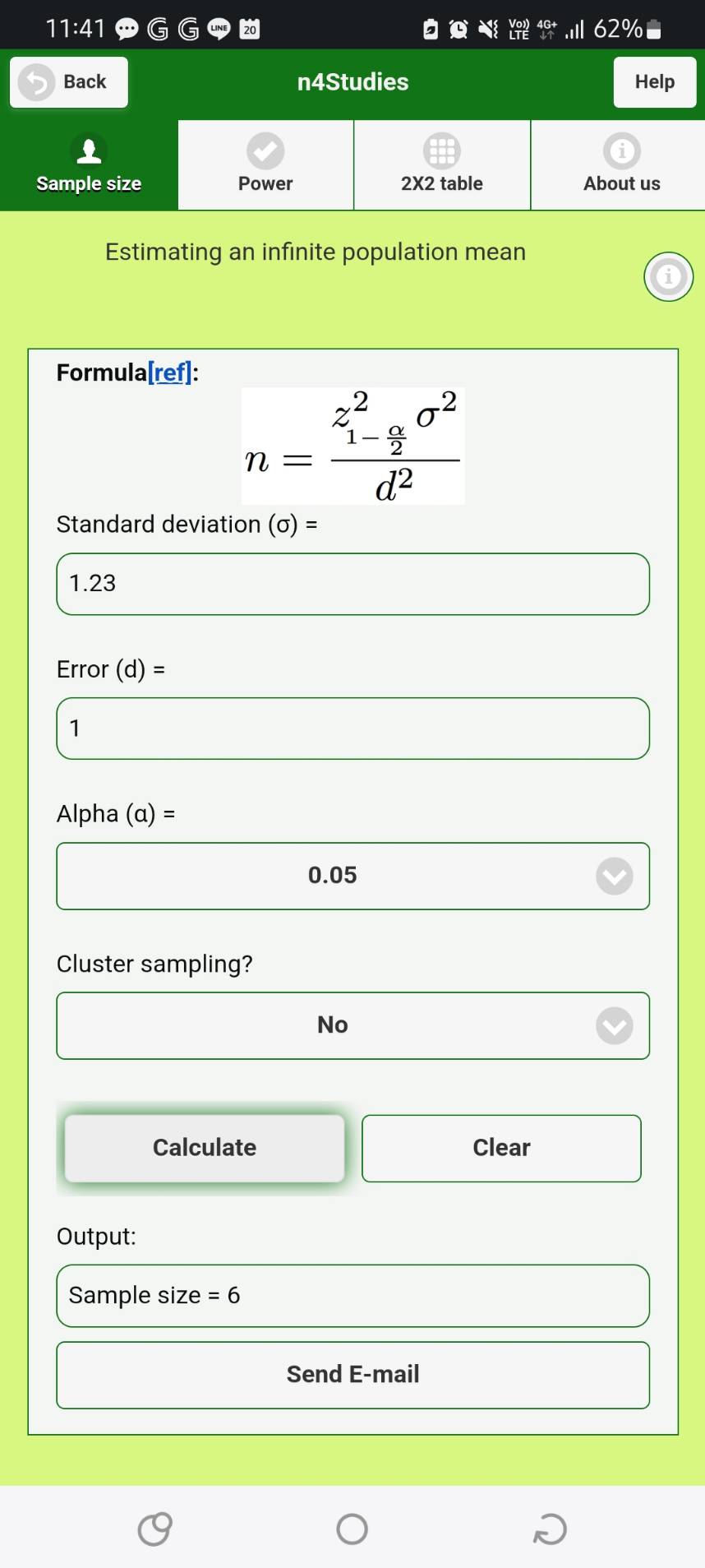


Here, σ = standard deviation, which was equal to 29.7 mg· h/liter. The precision (d) was assumed to be 15% of the mean which was equal to 26 mg· h /liter, $Z_{1-\frac{\alpha}{2}}^{2}$ = 1.96 using 95% confidence interval. Therefore, the minimum estimated sample size was 6 patients. Therefore, the study plan to inflate the sample size to 8 patients to cover 20% dropout. The 20% dropout of 8 patients would equal to 1.6, approximately 2 patients.
