# Supplementary material for: Dialysate and plasma meropenem concentrations in continuous intraperitoneal regimen during peritoneal-dialysis-related peritonitis
Source: PLoS One. 2025 Jan 6;20(1):e0312160. doi: 10.1371/journal.pone.0312160 (PMC11703071; doi:10.1371/journal.pone.0312160)
Supplement: S2 File — (DOCX) [file pone.0312160.s002.docx]

**Research Protocol**

**Study Title**

“Dialysate and Plasma Meropenem Concentrations in Continuous Intraperitoneal Regimen during Peritoneal-Dialysis-Related Peritonitis”

**Study Investigator(s)**

**Principal Investigator:** Thatsaphan Srithongkul (first author)

**Email:** thatsaphan.sri@mahidol.ac.th

**Co-Investigator:** Sukit Raksasuk

**Co-Investigator:** Bulaporn Techajongnumchai

**Co-Investigator:** Suchai Sritippayawan

**Co-Investigator:**  Pornpan Koomanachai (corresponding author)

**Email:**  nokmed@yahoo.com

**Institution:** Infectious Diseases and Tropical Medicine division,

Siriraj Hospital, Mahidol University

2 Wanglang Road, Bangkok Noi,

Bangkok, Thailand, 10700..

**Rationale & background**

Peritonitis, one of the most common complications of peritoneal dialysis (PD), leads to technical failure, increased hospitalization, and increased mortality among PD patients (1, 2). It has been estimated that 20%–30% of PD-related peritonitis is caused by gram-negative organisms, which are associated with worse outcomes (3, 4). Enterobacteriaceae are increasingly becoming resistant to many antibiotics regarding extended-spectrum β-lactamases (ESBLs)producing (5, 6). PD-related peritonitis involving ESBL-producing gram-negative strains has demonstrated poorer clinical outcomes, including an increased risk of treatment failure (7). ESBL-producing strains are usually susceptible to carbapenems and aminoglycosides. The rising number of multidrug-resistant organisms is resulting in the growing use of meropenem (8). The International Society for Peritoneal Dialysis (ISPD) has recommended the intraperitoneal (IP) antibiotic route for PD-related peritonitis in view of the higher drug concentrations that are made available at the infection site than with the intravenous route (1). However, data relating to IP meropenem dosing is still limited to only a few studies. The recommended dosage for use in continuous ambulatory peritoneal dialysis (CAPD) is a once-daily IP infusion of 1 g of meropenem, which is allowed to dwell for at least 6 hours (1). Nevertheless, this recommended dose is based on the pharmacokinetics performed on only one patient with Enterobacter cloacae peritonitis. The research concerned demonstrated that an IP meropenem dose of 1 g administered once daily achieved meropenem plasma levels exceeding the minimal inhibitory concentration (T > MIC) close to 100% of the time; unfortunately, no data for the dialysate drug concentration were reported (9). Another study by Wiesholzer and colleagues investigated pharmacokinetic data relating to a single dose of meropenem in automated peritoneal dialysis patients without peritonitis (10). The regimen was 500 mg of IP meropenem, which was allowed to dwell for 15 hours. A mean target value of 40% T > MIC (with a European Committee on Antimicrobial Susceptibility Testing susceptibility breakpoint of 2 mg/L) was achieved in serum and dialysate after the IP administrations (10). Nonetheless, the drug-dosing regimen utilized for automated peritoneal dialysis may not be applicable to CAPD, given the differences in their dialysate volumes and dwell times. Another point is that the study by Wiesholzer et al. investigated the meropenem levels in patients without peritonitis. Inflammation may have caused an increase in the peritoneal transport rate, so the pharmacokinetics of meropenem use in peritonitis patients may differ (11).

IP antibiotics can be given as continuous (every exchange) or intermittent (once-daily) doses, with each achieving similar outcomes in terms of treatment success and relapse rates (12). However, earlier studies have demonstrated that the intermittent IP administration of some beta-lactam antibiotics (such as cephalothin and ceftazidime) is associated with subtherapeutic drug levels in both plasma and dialysate, causing concern about the possibility of treatment failure(13, 14). Similarly, the efficacy of meropenem depends on achieving an adequate %T > MIC (a bactericidal target of  40%T > MIC) (15). The concentration of meropenem should be maintained at 2 to 4 times that of MIC throughout the dosing interval. The Clinical and Laboratory Standards Institute determined that the MIC breakpoints of meropenem against Enterobacteriaceae are ≤ 2 mg/L for susceptible microbes, 4 mg/L for intermediate microbes, and ≥ 8 mg/L for resistant microbes (16).

Therefore, a continuous administration of IP meropenem may provide a more adequate %T > MIC than an intermittent regimen. However, pharmacokinetic data for continuous IP meropenem administrations are still limited. There has only been a single case report which involved a CAPD patient with ESBL-producing Escherichia coli peritonitis. The patient received a continuous IP administration of meropenem at 125 mg/L for four cycles daily. The result was that the plasma meropenem concentrations at steady-state and the mean dialysate concentration were higher than the MIC for the resistant organisms (17). The study aim to investigate the efficacy of a continuous IP application in providing an adequate level of plasma and dialysate meropenem in CAPD patients with peritonitis.

**Study goals and objectives**

To measure blood and dialysate meropenem level after continuous intraperitoneal meropenem administration in CAPD patients with peritonitis.

**Study design**

Prospective descriptive study. The study will be conducted at Peritoneal dialysis unit, Siriraj Hospital, Mahidol University.

The previous study demonstrated the mean of plasma meropenem level from intraperitoneal meropenem was 7.23+1.2375 mg/liter with standard deviation 1.2375

$$\text{n} = \frac{\text{Z}_{\text{1-α}}^{2}\text{σ}^{2}}{\text{d}^{2}}$$

$$\text{n} = \frac{\text{(1.96)}^{2}\text{(1.2375)}^{2}}{\text{(1)}^{2}}$$

n = sample size

Z = statistic for level of confidence, using a 95% confidence interval (so Z = 1.96)

α = 0.05

σ = Standard deviation of the mean plasma meropenem level

d = precision

The estimated sample size using a power of 0.95 and 95% confidence interval was 6 patients. Therefore, The study plan to enroll 8 patients (+20% drop off).

**Duration of study:** 12 months

**Population:** The inclusion criteria for study enrolment are being aged 18 years or older and have achieved stable CAPD for at least one month prior to enrolment. The study will enroll all patients who is diagnosed with PD-related peritonitis as per the ISPD guidelines of 2016, which require that at least two of the following criteria be met: clinical features consistent with peritonitis (i.e., abdominal pain and a cloudy dialysis effluent); a dialysis-effluent white blood cell count of > 100/mm3, with more than 50% polymorph nuclear leukocytes (after a dwell time of at least 2 hours); and a positive dialysis-effluent culture.

The exclusion criteria are a previous antibiotic treatment during the 30 days preceding the provisional enrolment; advanced cirrhosis (Child-Pugh class C); a concomitant exit site or tunnel infection; peritonitis from other known causes; the presence of systemic inflammatory response syndrome; hemodynamic instability; concomitant antibiotic treatment for other conditions; and hypersensitivity or allergy to meropenem.

**Methodology**

Initially, complete drainage of the peritoneal dialysate will be performed in each patient. Subsequently, 2 L of dextrose dialysate are dwelled for 6 hours per exchange, with four exchanges daily. All patients will receive an IP meropenem loading dose of 500 mg in the first bag, then follow by maintenance therapy of 125 mg/L of IP meropenem in each exchange for four cycles daily (Figure 1). The dextrose concentration during the treatment will be adjusted individualized by peritoneal ultrafiltration, residual urine, and volume status.

The baseline demographic data collected consist of age, gender, body weight, height, body mass index, body surface area, the etiology of end-stage renal disease, comorbidities, peritoneal dialysis vintage, and current medications.

In the case of patients with a residual renal function, the urine volume and urine concentration are measured from 24 hours of urine samples. The residual glomerular filtration rate will be determined using the means of the urine creatinine clearance and the urine urea clearance, as follows:

• Creatinine clearance (ml/min) = (urine creatinine [mg/dl] x 24-hour urine volume [ml]) / (serum creatinine [mg/dl] x 1440) ----------------------------- (1)

• Urea clearance (ml/min) = (urine urea [mg/dl] x 24-hour urine volume [ml]) / (blood urea nitrogen [mg/dl] x 1440) ----------------------------- (2)

Before the administration of the IP meropenem, blood and dialysate samples are obtained as a baseline to verify the absence of any analytical interference from endogenous or exogenous sources. Time 0 (0 h) is defined as the time after complete drainage of the dialysate and before the meropenem administration. The blood samples (6 ml) are collected in lithium-heparin tubes for measurements of the plasma meropenem levels at baseline and 1, 2, 4, 12, and 24 hours following the first dose of meropenem administration. The dialysate samples (10 ml) are collected to determine the meropenem levels at baseline, and at 30 minutes, and 1, 4, 6, 12, 18, and 24 hours after the first meropenem application (Figure 1). The dialysate samples are obtained by draining 200 ml of dialysate into the empty bag attached to the patient before aspirating 10 ml of dialysate for analysis. The remaining dialysate is reinstalled into the peritoneal cavity. The total volume of each drained dialysate will be recorded.

All patients were followed up on Days 2, 5, and 14 after treatment. Dialysate samples are sent for a cell count, differential count, and culture to evaluate the treatment response. Successful treatment is defined as improved clinical features of the peritonitis, coupled with a dialysis-effluent white blood cell count of less than 100/mm^3^—with less than 50% polymorph nuclear leukocytes—within five days. The antibiotics and treatment durations will be adjusted according to the culture results. The antibiotic regimen will be adjusted depending on the physician's decision. If those results are negative, the study drug will be continued for a further 14 treatment days. All patients will be followed up on Day 14 after the commencement of the treatment to further evaluate their clinical responses.

The meropenem levels of the plasma and dialysate samples are quantified using high-performance liquid chromatography (HPLC). All HPLC analyses are carried out by a Waters Alliance liquid chromatography system (Waters Corporation, Milford, MA, USA), comprised of a Model 2695 Separate Module and a Model 2487 Dual Wavelength UV detector. HPLC separation was performed on a Symmetry C18 analytical column (5 µm, 250 x 4.6 mm I.D.), preceded by a sentry guard column C18 (5 µm, 20 x 3.9 mm I.D.; Waters Corporation, Milford, MA, USA), at a column temperature of 37°C (Temperature Control System, Waters Corporation, Milford, MA, USA). The mobile phase consisted of 0.5% of tetrabutylammonium hydroxide solution (25% in water), acetonitrile, and methanol (75:15:10, v/v), adjusted to pH 7.5 ± 0.1 with phosphoric acid. The mobile phase was filtered through an 0.2-µm membrane and degassed prior to use. The UV detection wavelength was 300 nm, and the flow rate was 1.3 mL/min. The analysis time is set at 10 min per sample, and the injection volume was 30 µL. The EmpowerPro software (Waters Corporation, Milford, MA, USA) is used to generate the standard curve by plotting areas under the curve of the extracted spike plasma versus various concentrations of meropenem.

The HPLC method is validated for its linearity, precision, accuracy, the limit of detection, and limit of quantitation, as per the International Conference on Harmonization guidelines. The meropenem calibration curve concentration range is 0.5–100 µg/mL. The linear equation of the calibration curve was y = 114.67x – 4.0454, and the linear correlation coefficients are 0.989 and 0.992 for plasma and peritoneal fluid, respectively. The intraday and interday precisions of meropenem are evaluated using three concentrations (1, 10, and 50 μg/mL), and the coefficient of variation (%CV) is lower than 2%. The method accuracy was determined using a recovery study conducted at three different concentrations (1, 10, and 50 μg/mL), and the average recoveries are 99.7 ± 1.2% and 99.3 ± 2.8% for the plasma and peritoneal fluid, respectively. In addition, the limits of detection for meropenem chromatographic determination are 0.5 and 1 μg/mL for the plasma and peritoneal fluid, respectively. Finally, the limits of quantitation for meropenem chromatographic determination are 1 and 2 μg/mL for the plasma and peritoneal fluid, respectively.

The blood samples (6 mL at each time point) are collected in the lithium-heparin tubes and centrifuged at 3,500 rpm for 10 min at 4°C. The blood cells are discharged, and the plasma samples are collected. Thereafter, the plasma and dialysate samples are divided into two aliquots of approximately 1 mL each before being stored at -80°C pending analysis. The meropenem concentrations of the plasma and dialysate are established using the HPLC method. A 400-μL aliquot of thawed clinical peritoneal fluid or plasma sample is transferred to a Nanosep 10K centrifugal filter device (Pall Life Sciences, Ann Arbor, MI, USA) and then centrifuged at 12,000 ×g for 10 min at room temperature. After the centrifugation, ~100 μL of the filtrate is collected in the filtrate reservoir of the device, and 30 µL of the supernatant is subsequently injected into the HPLC machine. The calibration curve of the meropenem is used to calculate the meropenem concentrations in the samples from their areas under the curve.

Figure 1 Schematic diagram of venous blood sampling and dialysate meropenem concentrations.


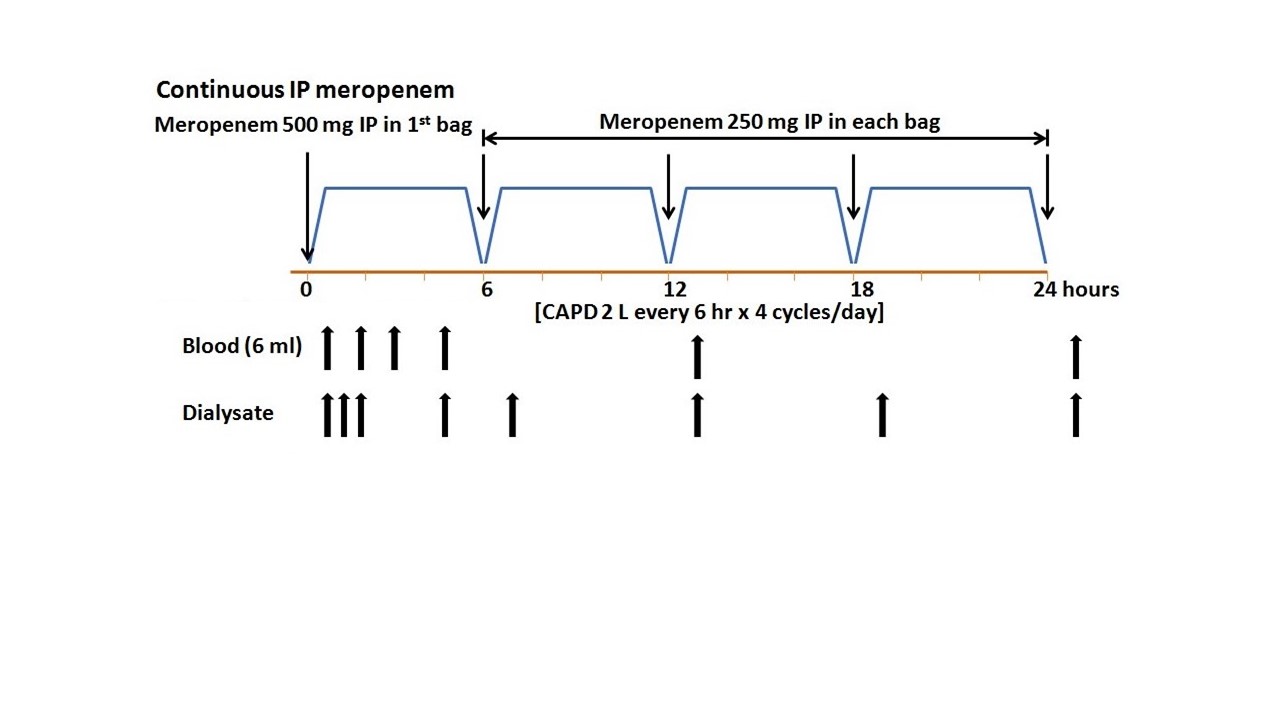


**Safety considerations**

The participant with a history of carbapenem allergy and unstable hemodynamics will be excluded. The participant will be admitted for 48 hours after enrollment to follow up on clinical response and adverse effects. All patients will be followed up on days 2, 5, and 14 after treatment for clinical and PD fluid cell count. The study protocol will be terminated in a participant who meets criteria as following criteria i) participant develops major side effects of meropenem such as drug allergy, seizure, or difficulty breathing. ii) participant hemodynamic is unstable or present with clinical sepsis. The participant with study termination will be evaluated promptly and receive standard treatment.

**Follow-up**

All patients will be followed up on Days 2, 5, and 14 after treatment for clinical, adverse events, and dialysates collection. Dialysate samples were sent for a cell count, differential count, and culture to evaluate the treatment response.

**Data management and statistical analysis**

These data will be collected using the instruments and methods described in the proposal. Records of results will be labeled and will be stored as hard copy and electronic files. All members of the investigative team can access to data. Original data notebooks will be retained in a secure location in the PI’s laboratory. All data will be kept for 3 years. To preserve confidentiality, each subject will be assigned an arbitrary code that will be associated with the data, and only data stripped of all potential identifiers will be stored in the collected and curated data sets. One file that contains the links to subject names and identifiers will be kept in a password-controlled file that will be accessible only to the subject coordinator. De-identified electronic data will be stored on external hard drives and DVDs, and copies of de-identified data will be preserved off site on the nephrology division server.

Categorical variables were presented as frequency and percentage. Descriptive analyses were presented as mean and standard deviation (SD). The plasma and dialysate meropenem levels were presented as a range, mean (SD), and median.

**Expected outcomes of the study**

Optimal dose of intraperitoneal meropenem for continuous intraperitoneal regimen.

**Ethics**

Participant who meet the criteria for enrollment will be informed detail of research protocal by investigator team. A subject voluntarily confirms his or her willingness to participate in a particular trial, after having been informed of all aspects of the trial that are relevant to the subject’s decision to participate.  Competent subjects able to comprehend the research-related information should personally decide and provide the consent on research participation.  Ethics approval was obtained from the Siriraj Institutional Review Board, Faculty of Medicine, Siriraj Hospital, Mahidol University (EC number 506/2560).

**Informed consent forms**

The informed consent form is written in Thai, that easily understood by the subjects. The subject will be given sufficient time to consider participation. Subject need understanding of the research and its risks, and it is tightly described in ethical codes and regulations for human subject research.

**Budget**

The study will be suppoeted by Routine to Research unit, Siriraj Hospital, Mahidol University.

**References**

1. Li PK-T, Szeto CC, Piraino B, de Arteaga J, Fan S, Figueiredo AE, et al. ISPD Peritonitis Recommendations: 2016 Update on Prevention and Treatment. Perit Dial Int. 2016;36(5):481-508.

2. Ye H, Zhou Q, Fan L, Guo Q, Mao H, Huang F, et al. The impact of peritoneal dialysis-related peritonitis on mortality in peritoneal dialysis patients. BMC Nephrol. 2017;18(1):186-.

3. Jarvis EM, Hawley CM, McDonald SP, Brown FG, Rosman JB, Wiggins KJ, et al. Predictors, treatment, and outcomes of non-Pseudomonas Gram-negative peritonitis. Kidney Int. 2010;78(4):408-14.

4. Szeto CC, Chow VC, Chow KM, Lai RW, Chung KY, Leung CB, et al. Enterobacteriaceae peritonitis complicating peritoneal dialysis: a review of 210 consecutive cases. Kidney Int. 2006;69(7):1245-52.

5. Bradford PA. Extended-spectrum beta-lactamases in the 21st century: characterization, epidemiology, and detection of this important resistance threat. Clinical microbiology reviews. 2001;14(4):933-51.

6. Wong SS, Ho PL, Yuen KY. Evolution of antibiotic resistance mechanisms and their relevance to dialysis-related infections. Perit Dial Int. 2007;27 Suppl 2:S272-80.

7. Yip T, Tse KC, Lam MF, Tang S, Li FK, Choy BY, et al. Risk factors and outcomes of extended-spectrum beta-lactamase-producing E. coli peritonitis in CAPD patients. Perit Dial Int. 2006;26(2):191-7.

8. Feng X, Yang X, Yi C, Guo Q, Mao H, Jiang Z, et al. Escherichia coli Peritonitis in peritoneal dialysis: the prevalence, antibiotic resistance and clinical outcomes in a South China dialysis center. Perit Dial Int. 2014;34(3):308-16.

9. Vlaar PJ, van Hulst M, Benne CA, Janssen WM. Intraperitoneal compared with intravenous meropenem for peritoneal dialysis-related peritonitis. Perit Dial Int. 2013;33(6):708-9.

10. Wiesholzer M, Pichler P, Reznicek G, Wimmer M, Kussmann M, Balcke P, et al. An Open, Randomized, Single-Center, Crossover Pharmacokinetic Study of Meropenem after Intraperitoneal and Intravenous Administration in Patients Receiving Automated Peritoneal Dialysis. Antimicrob Agents Chemother. 2016;60(5):2790-7.

11. Chung SH, Heimburger O, Stenvinkel P, Bergstrom J, Lindholm B. Association between inflammation and changes in residual renal function and peritoneal transport rate during the first year of dialysis. Nephrol Dial Transplant. 2001;16(11):2240-5.

12. Ballinger AE, Palmer SC, Wiggins KJ, Craig JC, Johnson DW, Cross NB, et al. Treatment for peritoneal dialysis-associated peritonitis. Cochrane Database Syst Rev. 2014(4):CD005284.

13. Roberts DM, Ranganathan D, Wallis SC, Varghese JM, Kark A, Lipman J, et al. Pharmacokinetics of Intraperitoneal Cefalothin and Cefazolin in Patients Being Treated for Peritoneal Dialysis-Associated Peritonitis. Perit Dial Int. 2016;36(4):415-20.

14. Booranalertpaisarn V, Eiam-Ong S, Wittayalertpanya S, Kanjanabutr T, Na Ayudhya DP. Pharmacokinetics of ceftazidime in CAPD-related peritonitis. Perit Dial Int. 2003;23(6):574-9.

15. Nicolau DP. Pharmacokinetic and pharmacodynamic properties of meropenem. Clin Infect Dis. 2008;47 Suppl 1:S32-40.

16. Clinical and Laboratory Standards Institute. Performance Standards for Antimicrobial Susceptibility Testing: Twenty-sixth Informational Supplement M100-S27. CLSI, Wayne, PA, USA, 2017.

17. de Fijter CW, Jakulj L, Amiri F, Zandvliet A, Franssen E. Intraperitoneal Meropenem for Polymicrobial Peritoneal Dialysis-Related Peritonitis. Perit Dial Int. 2016;36(5):572-3.

18. Convention USP. USP 29, NF 24: the United States Pharmacopeia, the National Formulary. Gilbert BD, editor: United States Pharmacopeial Convention; 2005.
