# Supplementary material for: Dialysate and plasma meropenem concentrations in continuous intraperitoneal regimen during peritoneal-dialysis-related peritonitis
Source: PLoS One. 2025 Jan 6;20(1):e0312160. doi: 10.1371/journal.pone.0312160 (PMC11703071; doi:10.1371/journal.pone.0312160)
Supplement: Attachment — Submitted filename: Thatsaphan et al._response to reveiwer.docx [file pone.0312160.s004.docx]

Dear Reviewers,

Many thanks for your constructive feedback and suggestions. We have addressed all of the issues below and in the manuscript. We believe these changes have improved the manuscript considerably and hope that it is now acceptable for publication.

**In response to Editor:**

1.Within the introduction, there is a need to further justify the dosing regimen chosen. Was this based on general practice (e.g. usual dosing at your center) or is there other data relied upon to determine the dosing regimen?

Thank you for your comment. According to previous research from Fitjer et al. demonstrated that IP meropenem 125 mg/L for 4 exchanges daily provided adequate plasma and dialysate meropenem level and safety profiles. According to ISPD guidelines for peritonitis recommended loading dose in continuous regimen of IP route to achieve therapeutic drug level, so we selected to use loading dose of 500 mg meropenem for initial dose. This explanation has been added in text.

2.Further explanation of the 24h dialysate levels are required. It would appear from the data presented that the mean (SD) at 24h was 32.6 (19): this dispersion would suggest that there is not confidence that the mean would consistently be 4x greater than MIC's for resistant organisms (e.g. >8 mg/L).

Thank you for your suggestion. Unfortunately, we did not have the data of drug level after 24 hours. However, the meropenem IP will be added 125 mg/L every 6 hours in continuous IP regimen, that will maintain the meropenem level in dialysate. Furthermore, we did not expect to use meropenem to treat CRE (MIC >8mg/L). If CRE was suspicious, we would rather use other ATB such as colistin. This text has been added in discussion section.

3. Please provide further comment on the excretion pharmacokinetics of meropenem given that the plasma levels at 24 h were still increasing in 3/5 participants (Figure 4B). The statement that evidence of adverse effects related to meropenem should be removed given the short duration of this study, and the study did not include a protocol for examining treatment related adverse effects.

Thank you for your comment. The plasma level of meropenem at 24 hour was increased due to the third dose of IP meropenem at 18 hr. Similarly, P.J. Vlaar reported the highest concentration of plasma meropenem was observed after 4 hours of IP meropenem administration.

The statement of adverse effects related to meropenem has been removed as suggestion.

4. The discussion should be more specific that the potential use of IP meropenem with this dosing regimen would be restricted to individuals without bacteremia, or evidence of (severe) sepsis. The term 'septicemia' should be avoided.

Thank you very much. the text of “Therefore, the present regimen should be restricted in the patient with PD related peritonitis, who did not have signs and symptoms of systemic bacteremia” has been added in the text.

**In response to Reviewer 1:**

1. As this isn't a randomised trial figure 1 could usefully just be called a flowchart as CONSORT is for randomised trials.

Thank you. The legend of Figure1 has been changed to “patient enrollment flow chart” in the text.

2. The sample size needs to be explained more fully - the first sentence I think is missing a word such as estimate. A sample size like 6 is too small for the standard sample size formulae as the approximations are not good for small numbers - as such please give more details on the calculation. Is the "+20% dropoff" an increase of 20% to allow for 16% dropout or an inflation of 25% to allow for 20% dropout? Are the numbers in this section with +/- symbols sd or sem?

Thank you for your suggestion. According to our study design, which was a descriptive study to evaluate the level of meropenem in dialysate, and for this reason there is no existing literature with which to perform a power calculation. Therefore, the section of power calculation was not necessary and has been removed in the text.

3. Please remember that for normality double the standard deviation needs to be less than the mean. As such the reporting of mean and sd is not always a fair measure of the distribution.

Thank you for your advice. Median and IQR has been added in the reporting of non-normal distribution data.

4. At what point did the 2 patients drop out - were any post entry datapoints collected? How did these look as we know these are not responders and as such are particularly of interest.

Thank you for your comment. One patient developed clinical of SIRS and hypotension at 2 hours after loading dose of intraperitoneal meropenem and another patient was diagnosed with secondary peritonitis after first dose of intraperitoneal meropenem, so the route of antimicrobial was changed to intravenous route. The explanation has been added in the text.

5. The legend on figure 3a does not tell us what the error bard represent. Ditto for figure 4a.

Thank you. The bared represent mean ± SD. The legend of figure 3a and 4a was revised in the text.

**In response to Reviewer 2:**

1.Firstly I think the authors should clearly document how this manuscript adds to current literature that their reference 17 already describes.

Thank you for your comment. According to previous study from Fitjer et al. demonstrated that IP meropenem 125 mg/L for 4 exchanges daily provided adequate plasma and dialysate meropenem level. However, the study was done in only one patient and IP meropenem was administered after intravenous antimicrobial. Therefore, our study aimed to evaluate meropenem level from continuous regimen of IP meropenem in more patients. The explanation has been added in the article.

2. I don’t see why the authors use a subheading in line 208 mentioning sample size calculation? The following description does not justify their sample size of five patients.

Thank you for your comments. According to the study design which was a descriptive study to evaluate the level of meropenem in dialysate, and for this reason there is no existing literature with which to perform a power calculation. Therefore, the section of power calculation was not necessary and has been removed in the text.

3.The authors statement in line 263 of “proven” should rather say “documents”…ie ……The present study has proven that a continuous IP meropenem regimen…I suggest ….The present study documents that a continuous IP meropenem regimen….

Thank you for your suggestion. The statement has been changed follow your recommendation.

4.Ref 5 should have a journal abbreviation

Thank you. The journal abbreviation has been replaced in reference 5.

5. Can the authors please comment on the second spike in levels of patient 3 and patient 4 in figure 3b

Thank you for your comment. The second spike in dialysate levels in patient 3 and 4 at 4 hours and 6 hours may be explained by directional of solute transport through peritoneal membrane that caused diffusion of drug from plasma to dialysate.

**In response to Reviewer 3:**

The authors report on the dialysate and plasma meropenem concentrations in CAPD patients.

1. The study consists of 5 patients, which is usually normal for a pharmocokinetic study. However, the patient population is far from uniform in this example. The majority of patients do not even have gram (-) bacteria. As the authors state, one has fungal, one has streptococci and the two are culture negative. This makes the response rates to meropenem irrelevant.

Thank you for your comment. I agree that from our present article could not proved the efficacy of IP meropenem due to the population in our study did not have gram negative bacteremia. However, the primary objective of the study was to evaluated plasma and dialysate meropenem level from IP meropenem during peritonitis, which may interfere drug bioavailability and absorption. The further study is needed for proven efficacy of IP meropenem in PD related peritonitis from gram negative bacteria. This explanation has been stated in the text.

2. Even though there has been multiple blood and dialysate sampling, there is no PK data or compartment modeling using a software program incorporating the pharmacokinetic parameters.

Thank you for your comment. Unfortunately, we did not have pharmacokinetic data in our study. The further study with PK data is needed to evaluate efficacy of present regimen of meropenem. This text has been added in limitation section.

3. According to this study, all the levels achieved are way over 8mg/L so all this study tells me is that the dose used in the study was too much.

I think the authors should use this study and data as a preliminary study and plan another one with a uniform patient population and different dosing regimen, incorporating the parameters into the system.

Thank you for your suggestion. I agree that further study is needed for evaluating pharmacokinetic and treatment outcome and the text has been added in the limitation of study.

**In response to Reviewer 4:**

I wish to thank the authors for this submission on an important topic, that is treating resistant infections in PD peritonitis. This is important work, and appears to be a shortcoming in the existing literature.

1. Power calculation - you have performed a descriptive, proof of concept study and for this reason there is no existing literature with which to perform a power calculation. I also do not think it is necessary, although warrants toning down the assertion that this is an effective treatment for PD peritonitis. Your study demonstrates that therapeutic levels of meropenem can be achieved in the dialysate, and now further studies to assess the efficacy, for example in RCT, would be warranted.

Thank you for your suggestions. We agreed that power calculation did not necessary in our study that aimed to evaluate therapeutic levels of meropenem. Therefore, the section of power calculation has been removed in the text.

2. Pharmacokinetics-it may be valuable to explain to the readers the pharmacokinetics of meropenem. This background would help explain why administration via the PD route is feasible, and I would suggest including parameters such as the volume of distribution, protein binding and route of excretion. It would also be helpful to explain any of the variables with peritoneal dialysis that affect pharmacokinetic properties such as absorption, distribution and excretion. With the discrepancies you see between individuals, it may be important to describe the characteristics of their dialysis. I wondered about the dextrose concentration and if this plays a role in the movement across the peritoneal membrane. Additionally, does residual renal function play a role? If meropenem undergoes tubular excretion, is all residual renal function equal?

Thank you for your suggestions. Meropenem is beta-lactam antibiotic, which demonstrated low to moderate volume of distributions and low protein binding. Meropenem molecules is small and hydrophilic. Since meropenem is primarily eliminated unchanged in the urine, accumulation of the drug was seen in patients with renal impairment. Previous report from Wiesholzer et al. demonstrated that IP meropenem provided adequate dialysate drug level better than intravenous route. For PD related peritonitis treatment required adequate antimicrobial dosing at site of infection, so IP route of meropenem is feasible and preferred in PD related peritonitis from ESBL producing gram negative bacteria.

The other explanations for discrepancies between individuals, may be explained by different residual renal function, peritoneal solute transport rate and peritoneal surface area. Whitty R. et al. reported treatment failure was increased in patients with urinary creatinine clearance more than 5 mL/min. However, previous study from Heimbürger O et al. demonstrated that the diffusive transport of small solutes through the peritoneal membrane is not influenced by the initial concentration of glucose. The explanation has been added in the text.

3. Discussion - As mentioned previously, I think additional studies to demonstrate efficacy would be warranted, as you have shown what appears to be effective dialysate concentrations. Are there other variables that explain the differences in meropenem concentrations beyond inter-individual variability? It would be helpful to consider both the pharmacokinetic considerations described above, but also potential disease characteristics that play a role. For example, patient 4, is there something unique about fungal peritonitis that makes it more permeable or pro inflammatory there by decreasing the concentration gradient observed and resulting in a more uniform concentration between the two compartments? Further to these pharmacokinetic considerations above, would you advocate for measuring meropenem concentrations in the urine for patients with residual renal function?

Thank you for your comments. Other factors that played a role of the difference in meropenem concentration would be different residual renal function, peritoneal membrane transport and peritoneal surface area. Patients with significant residual renal function, meropenem concentration in urine should be measured for pharmacokinetics consideration in the future research.
