# Supplementary material for: Dialysate and plasma meropenem concentrations in continuous intraperitoneal regimen during peritoneal-dialysis-related peritonitis
Source: PLoS One. 2025 Jan 6;20(1):e0312160. doi: 10.1371/journal.pone.0312160 (PMC11703071; doi:10.1371/journal.pone.0312160)
Supplement: Attachment — Submitted filename: Thatsaphan et al._ 2nd reviewer response.docx [file pone.0312160.s005.docx]

**Dear Reviewers,**

Many thanks for your constructive feedback and suggestions. We have addressed all of the issues below and in the manuscript. We believe these changes have improved the manuscript considerably and hope that it is now acceptable for publication.

**In response to Reviewer 1:**

Reviewer #1: The sample size as this is a prospective study is required and a valid justification of it including dropout which was not handled clearly in the original version of the paper. It is not scientifically valid to remove this section as it now misleads the reader as to the planned intent.

**Thank you for your comment. We acknowledged that sample size calculation and detail is important for most prospective studies. We have now added the sample size calculation in the supplement. There, we also addressed our rationale of the adjustment for sample size dropout as well.**

**In response to Reviewer 2:**

The authors have adequately addressed all the comments I had made. Whilst I think they have addressed other reviewer comments, the other reviewers should comment.

**Thank you for your constructive feedback.**

**In response to Reviewer 3:**

Unfortunately, my previous comments are still relevant such as the lack of a uniform study population as well as pharmacokinetic data and very high meropenem levels throughout the study. Even though the idea of this manuscript is sound and has merit, the study itself needs further work.

**We appreciate your feedback on the uniformity of the study population and the concern of the drug level. We have added our rationale in the discussion to further clarify this. As we aimed to study the drug level of meropenem in an inflamed peritoneum, it is difficult to rationalize this type of study in patients with proven ESBL-gram negative bacteria which requires that the drug level has to be achieved. This study result can then be used as a foundational data for further study with specific enrollment for patients with ESBL-gram negative bacteria.**

**In regards to the high drug level, it is a great point that you have commented. However, we have not observed any signs of meropenem toxicity in our patient. Acknowledging that the drug level is higher than that what is needed, we have added in the discussion to suggest a change in the loading regimen in future studies to both minimize the risk of inadequate dosing and toxicity.**

**Unfortunately, the full pharmacokinetics profiles of the drug is beyond the scope of our work and we are not able to profile such data at this time. We have added this limitation in our discussion as well.**

**In response to Reviewer 4:**

1.The introduction would benefit with a proof read by a native English speaker for flow and clarity. There are a few sentences that should be rewritten, for example "Enterobacteriaceae are increasingly becoming resistant to many antibiotics regarding extended-spectrum β-lactamases (ESBLs)producing.

**Changes has been made to as follow. Thank you for your feedback. We have also sent the manuscript to the language support service of Mahidol University to further adjust the flow of the wording used in this manuscript.**

**“Enterobacteriaceae and extended spectrum Beta-lactamases (ESBLs) producing bacteria are becoming increasingly resistant to many antibiotics[****5****,** **6****]. PD related peritonitis involving ESBL-producing gram-negative strains have demonstrated poorer clinical outcomes, including an increased risk of treatment failure [****7****].”**

2.Additionally, I think it may be worth mentioning how much higher the levels are above the MIC targets and that future studies may include exploring dose reductions.

**Thank you for pointing out on the aspect of the level of meropenem. To clearly show ‘how much higher’, we have adjusted figure 4B (blood meropenem level) to include the MIC level in dash line. We have also included a statement about dose reduction in future studies in the discussion.**

3. Line 88 - Should that line read > 2 mg/L for susceptible organisms?

**Thank you so much for your hardwork here. We have adjusted accordingly.**

4. Line 98 - I think this study examines feasibility, not efficacy, efficacy would need to be outcomes based.

**We agree with your comment and has now adjusted the language to be a feasibility study.**

Line 222/292 - septicaemia should be removed and alternate terminology used (suspected bacteremia).

**Thank you for your suggestion. We have revised accordingly.**
