# Supplementary material for: Dialysate and plasma meropenem concentrations in continuous intraperitoneal regimen during peritoneal-dialysis-related peritonitis
Source: PLoS One. 2025 Jan 6;20(1):e0312160. doi: 10.1371/journal.pone.0312160 (PMC11703071; doi:10.1371/journal.pone.0312160)
Supplement: Attachment — Submitted filename: Thatsaphan et al_3rdResponse to reviewers.docx [file pone.0312160.s006.docx]

**Dear Editor and Reviewers,**

We do appreciate all of your suggestions and comments. We have addressed all of the issues below and in the manuscript. We believe these changes have improved the manuscript considerably and hope that it is now acceptable for publication.

**In response to Reviewer 1:**

1. The primary outcome was not "to evaluate ..." - this is the primary aim. The primary outcome is the variable used to measure this. Please clarify and state in the methods that the aim was to estimate the parameter with a certain degree of accuracy.

**Thank you for your comment. We have adjusted in the main text as your suggestion which has provided more clarify on the definition of the primary outcome.**

2. Please tie the variable used for the power calculation in with the numbers given in the sample size calculation which would appear to be AUC as opposed to the information given in the results.

**Thank you for raising up this point on the sample calculation. We added the sample size calculation based on previously published AUC_0–24_ of meropenem in serum reported by Wiesholzer et al. in the figure attached below and in the supplementary file (S1 file). Additionally, we have included the outcome regard to AUC_0–24_ of meropenem in plasma and dialysate as shown in the revised manuscript.**

3. The results likewise do not report a normal distribution which is what is assumed

**We agree that it is not straight forward to assume normal distribution in this sample. Following your recommendation, now we have added both mean ± SD and medium + IQR to ensure all dimensions of the data are represented.**

4. Please justify an infinite population assumption for 6 [patients in](http://patients.in/) a sample where one has to estimate the variance as well.

**Although we do not know the total target population, it is likely that the target population for our study is very large. Also, given the random sampling from a large pool of patients in our hospital, there should be a negligible sampling fraction. Given that prior study with similar goals has also provided the estimated variance, we felt that using the infinite population mean formula should be reasonable. The calculation is shown in the figure attached below.**
